# Novel Candidate Genes Differentially Expressed in Glyphosate-Treated Horseweed (*Conyza canadensis*)

**DOI:** 10.3390/genes12101616

**Published:** 2021-10-14

**Authors:** Yongil Yang, Cory Gardner, Pallavi Gupta, Yanhui Peng, Cristiano Piasecki, Reginald J. Millwood, Tae-Hyuk Ahn, C. Neal Stewart

**Affiliations:** 1 Department of Plant Sciences, University of Tennessee, Knoxville, TN 37996, USA; yyang98@utk.edu (Y.Y.); yhpeng@gmail.com (Y.P.); cristiano.piasecki@atsibrasil.com.br (C.P.); rmillwood@utk.edu (R.J.M.); 2 Center for Agricultural Synthetic Biology, University of Tennessee, Knoxville, TN 37996, USA; 3 Program in Bioinformatics and Computational Biology, Saint Louis University, St. Louis, MO 63103, USA; cory.gardner@slu.edu (C.G.); pg3fy@umsystem.edu (P.G.); taehyuk.ahn@slu.edu (T.-H.A.); 4 MU Institute for Data Science and Informatics, University of Missouri, Columbia, MO 65211, USA; 5 Centers for Disease Control and Prevention, 1600 Clifton Rd., Atlanta, GA 30333, USA; 6 ATSI Brasil Pesquisa e Consultoria, Passo Fundo 99054-328, RS, Brazil; 7 Department of Computer Science, Saint Louis University, St. Louis, MO 63103, USA

**Keywords:** *Conyza canadensis*, glyphosate, non-target-site-based resistance, differentially expressed gene analysis, membrane-bound protein kinase, CYP450, ABC transporter

## Abstract

The evolution of herbicide-resistant weed species is a serious threat for weed control. Therefore, we need an improved understanding of how gene regulation confers herbicide resistance in order to slow the evolution of resistance. The present study analyzed differentially expressed genes after glyphosate treatment on a glyphosate-resistant Tennessee ecotype (TNR) of horseweed (*Conyza canadensis*), compared to a susceptible biotype (TNS). A read size of 100.2 M was sequenced on the Illumina platform and subjected to de novo assembly, resulting in 77,072 gene-level contigs, of which 32,493 were uniquely annotated by a BlastX alignment of protein sequence similarity. The most differentially expressed genes were enriched in the gene ontology (GO) term of the transmembrane transport protein. In addition, fifteen upregulated genes were identified in TNR after glyphosate treatment but were not detected in TNS. Ten of these upregulated genes were transmembrane transporter or kinase receptor proteins. Therefore, a combination of changes in gene expression among transmembrane receptor and kinase receptor proteins may be important for endowing non-target-site glyphosate-resistant *C. canadensis*.

## 1. Introduction

Weeds continue to evolve in agronomic ecosystems, in many cases, to become more weedy and a bane to farmers. Herbicides have emerged as the major weed management strategy in many parts of the world. Among the various commercial herbicides, glyphosate has been regarded as an effective chemical herbicide because of its relatively low environmental risk and economic cost [1]. Glyphosate is an *N*-phosphonomethyl-modified derivative of glycine. After plants are treated, glyphosate forms a stable complex with the enzyme 5-enolpyruvylshikimate-3-phosphate synthase (EPSPS), thereby inhibiting the shikimate pathway in chloroplasts [2,3]. However, wide and intensive use of glyphosate has led to the appearance of glyphosate-resistant weeds. Since rigid ryegrass (*Lolium rigidum*) was reported from Australia as the first glyphosate-resistant weed [4], 38 weed species have evolved resistance to glyphosate worldwide [5]. The appearance of glyphosate-resistance among weeds impacts both weed control costs and crop yields [6]. Among glyphosate-resistant weed species, horseweed (*Conyza canadensis*), and its congener, hairy fleabane (*Conyza bonariensis*), of the Asteraceae family, are regarded as more troublesome weeds because they have evolved to resist glyphosate [7].

There are several putative routes for glyphosate resistance in weedy plants: (1) gene mutation or duplication of the glyphosate target site in a specific protein, such as 5-enolpyruvilshikimate-3-phosphate synthase (EPSPS); (2) chemical modification of the active glyphosate structure; (3) active sequestration of glyphosate in the vacuole or prevention of cellular uptake of glyphosate; and (4) a hyperresponse to necrosis to handle toxicity caused by glyphosate [8]. Generally, the first category has been referred to as a target-site resistance (TSR) mechanism, while the other three are non-target-site resistance (*NTSR*) mechanisms [9]. In addition, glyphosate-resistant weeds could utilize several resistance mechanisms simultaneously, allowing effective glyphosate resistance. There have been more studies on the physiological mechanisms than on the molecular mechanisms. Molecular mechanisms differentiating glyphosate-resistant and -susceptible biotypes in a weed remain relatively underexplored.

Relatively few EPSPS coding mutations have been shown to endow resistance. Examples include single base changes in Pro106 to Ser, Ala, Thr, or Leu, usually resulting in two- to six-fold glyphosate resistance [10,11]

The gene expression level of EPSPS does not differ among the glyphosate-sensitive populations of *C. canadensis* and *C. bonariensis* after glyphosate treatment [12]. Furthermore, the single nucleotide polymorphism or differential gene expression of EPSPS has not been detected in two different high-throughput sequencing results of *C. bonariensis* [13,14]. These results indicated that EPSPS was unlikely to be a player in glyphosate resistance in *C. bonariensis*. Therefore, the different mechanisms using non-target-site resistance (*NTSR*)-related genes, including tonoplast-localized transporters or different target-site modifications of glyphosate-resistance-related genes, seem to be plausible hypotheses to explain glyphosate resistance.

Glyphosate resistance can be induced by a decrease in the absorption of glyphosate into the cell or through the sequestration of glyphosate into the vacuole. A reduced absorption of glyphosate was reported in the glyphosate-resistant biotypes of several weed species [15,16,17]. Furthermore, glyphosate resistance through the sequestration of glyphosate into the vacuole was observed in *C. canadensis* and *Lolium spp.* [18,19]. ^31^Phosphate-labeled glyphosate was predominantly sequestered into the vacuole of glyphosate-resistant *C. canadensis* [20], indicating that the tonoplast-localized transporter plays a role in glyphosate sequestration. Three different studies showed that M10 and M11 gene expression, encoding the ATP-binding cassette transporter subgroup C (ABC-C) of the tonoplast-localized transporter (ABC transporter), were induced dramatically by glyphosate treatment in *C. canadensis* [12,21,22]. In addition, *M10* and *M11* were also induced in glyphosate-treated *C. bonariensis* [13]. To date, the differential expression of ABC transporters in the glyphosate-resistant and glyphosate-susceptible biotypes of *C. bonariensis,* after glyphosate treatment, were summarized in two different transcriptome analyses [13,14]. Both studies determined that ABC transporters were transcribed within a relatively narrow range in both the glyphosate-suceptible and -resistant populations, hence this gene is also not regarded as a glyphosate-specific responsive mechanism [13]. Besides the EPSPS and ABC transporters, the related research of other glyphosate-resistant genes in *Conyza* has been scarce.

Illumina and Hi-C technologies have allowed the assembly of a more complete *C. canadensis* genome, allowing for more advanced biochemical approaches to weed control than before [23,24]. In addition, our lab performed a high-throughput transcriptome analysis of glyphosate-treated *C. canadensis* by the GS-FLX 454 pyrosequencing technique to investigate differentially expressed gene groups depending on glyphosate treatment [21]. We could predict 17 ABC transporter transcripts that have been regarded as representative *NTSR* genes in sequential research. However, we are still searching for more potential genes that are differentially regulated by glyphosate treatment. The present study introduces novel differentially expressed genes in glyphosate-resistant and -susceptible *C. canadensis* biotypes (TN ecotype). We propose several novel gene groups that may endow glyphosate resistance.

## 2. Materials and Methods

### 2.1. Plant Sample Preparation and RNA Extraction

*C. canadensis* accessions were collected from middle Tennessee, USA [24]. The collected seeds were grown in potting media at the University of Tennessee, Knoxville, TN, USA, under a 16/8 h of light/dark photoperiod at 25 °C. Glyphosate-resistant plants were screened by spraying glyphosate (Roundup WeatherMax; 0.84 kg acid equivalent ha^−1^; Monsanto, St. Louis, MO, USA). Glyphosate-susceptible (TNS) or -resistant (TNR) *C. canadensis* of the Tennessee biotype were watered and fertilized with Osmocote slow-release fertilizer. For glyphosate treatment, three plants from each of the six TNR or TNS biological replicates were grown in 1 L pots until rosette leaves reached a diameter of 6 to 8 cm (approximately 3-month-old plants). Rosette-stage plants were treated by spraying glyphosate. For the non-glyphosate treated (water-sprayed) TNS and TNR plants, three plants from each of the seven TNR, or eight TNS biological replicates, were used as experiment control samples. The aboveground biomass of the glyphosate-sprayed and water-sprayed TNS and TNR was collected 24 h after treatment, and quickly frozen in liquid nitrogen. Samples were stored at −80 °C before RNA extraction.

The frozen leaves were ground by mortar and pestle. Total RNA was extracted using TriReagent following the manufacturer’s protocol (Invitrogen, ThermoFisher Scientific, Carlsbad, CA, USA). Extracted RNA was characterized for purity and concentration with a Nanodrop spectrophotometer (ThermoFisher Scientific). The RNA quality was assessed again by RNA chip analysis on an Agilent 2100 Bioanalyzer system (Agilent, Santa Santa Clara, CA, USA). All RNA samples were treated with DNase I (Invitrogen, ThermoFisher Scientific) to remove residual genomic DNA.

### 2.2. cDNA Library Preparation and Illumina Sequencing

The Illumina libraries were prepared by using an RNA-Seq prep kit (Illumina Inc., San Diego, CA USA) according to the manufacturer’s protocol. A total of 27 libraries were constructed from total RNA from 7 TNR (TNRC) water-sprayed leaves, as well as those from glyphosate-sprayed 6 TNR (TNRG), water-sprayed 8 TNS (TNSC), and glyphosate-sprayed 6 TNS (TNSG) leaves. Each library was ligated with a unique index barcode. All cDNA libraries were sequenced by using a HiSeq2000 (Illumina Inc.) at Oak Ridge National Laboratory. The generated sequence reads were single-ended and 100 bp in length.

### 2.3. Read Quality Control and De novo Transcriptome Analysis

The read quality control (QC) of raw reads was performed by MultiQC [25]. The adaptor sequence and low-quality reads were removed by Sickle (https://github.com/najoshi/sickle; accessed on 10 March 2020). The trimmed sequences were subjected to de novo transcriptome analysis using Trinity software [26]. The whole transcriptome reference sequence was assembled from TNRC, TNRG, TNSC, and TNSG raw reads by the Trinity assembly function. The assembled transcript contigs were functionally annotated from five different databases (nr, nt, Pfam, Uniprot, and eggnog) by the Trinotate function. NCBI accession identities were then fetched by efetch distributed with BlastX [27].

The qualified reads were mapped and annotated against the whole transcriptome reference and protein annotations by the Trinity quantification script, using default parameters. Under this script, Bowtie2 was performed to align reads, and RSEM (RNA-Seq by Expectation-Maximization) was used for the quantification with maximum likelihood estimates [28,29]. The read matrices of gene-level and transcript-isoform-level data were generated by our own script for downstream analysis.

Principal component analysis (PCA) was performed and plotted by the prcomp and ggplot packages in R [30].

### 2.4. Differentially Expressed Gene (DEG) Analysis

The gene-level transcript read count matrix from the RSEM output was submitted to edgeR for DEG analysis [31]. A total of 24 transcript quantities of comparable combinations between glyphosate-treated TNR or TNS biotypes, and water-sprayed TNR or TNS, were subjected to DEG analysis. In addition, water-sprayed TNR and TNS were analyzed following the same process. The genes were filtered using the false discovery rate (FDR) criterion for each analysis. Statistically, the transcripts of up- or downregulation over 4 folds (2 of log2) with significant FDR (<0.05) were taken as differentially expressed genes.

### 2.5. Validation of Representative DEGs by Quantitative Reverse Transcription Polymerase Chain Reaction (RT-qPCR)

Total RNA samples were obtained by the RNA-extraction protocol as described above. One microgram of total RNA was reverse transcribed by the RevertAid First Strand cDNA Synthesis Kit (Thermo Fisher Scientific). One μL of three-times diluted cDNA was added to 15 μL of 1 × PowerUp SYBR Green Master Mix, as well as a pair of primers, as listed in Table S1 (Applied Biosystems, Waltham, MA, USA). After preheating at 50 °C for 2 min, and predenaturation at 95 °C for 10 min, two-step protocol sequencing at 95 °C for 15 s, and at 60 °C for 30 s, were performed. The real-time PCR reaction was operated by the QuantiStudio™ 6 Flex real-time PCR system (Thermo Fisher Scientific). The relative gene expression change was determined by the 2^−ΔΔ*Ct*^ equation [32]. Statistical analysis was performed with the relative fold change of three technical replicates from three different horseweed plants by a Student’s *t*-test.

### 2.6. Transcript Alignments and Prediction of Gene Identities

To find the transcript contigs of the ABC transporters in whole transcript assemblies, we downloaded the transcript sequence from the reference [21]. We aligned the sequences to a fasta file of the whole transcript assembly using Minimap2 [33]. Other transcript contigs of interest were also aligned with transcript sequence information from the NCBI database by the same procedure [27]. To verify the gene identities of those not assigned a protein function, we translated the open reading frame in the transcript contigs using the amino acid sequence translation tool at the ExPaSy website (https://web.expasy.org/translate/; accessed on 11 May 2020). The longest open reading frame was submitted to the blastp suite in NCBI (https://blast.ncbi.nlm.nih.gov/Blast.cgi; accessed on 11 May 2020) to identify the similar protein sequence in the protein database.

### 2.7. Calculation Correlation Efficiency and Plot Generation

The correlation efficiency was calculated using the Pearson method in R. All plots were generated by ggplot2 in R [30].

### 2.8. Gene Ontology (GO) Analysis

On the basis of the annotation file information, the NCBI protein accession number was matched for each gene. GO terms were assigned using the accession numbers provided by DAVID [34], with 24 significantly overrepresented GO terms based upon *Arabidopsis thaliana*’s definition (*p* < 0.05).

## 3. Results

### 3.1. Bioinformatic Information of Illumina Sequencing

Twenty-seven cDNA libraries were generated from TNR and TNS leaves 24 h after glyphosate or water treatment, and then submitted for high-throughput Illumina sequencing (Figure S1). A total of 100.2 million reads were sequenced (Table 1). The sequenced data were adapter-trimmed and quality-filtered. Through read quality control, we excluded three raw sequencing replicates because of their low recovery rates. The remaining high-quality reads from 24 libraries (6 TNRC; 5 TNRG; 8 TNSC; 5 TNSG) were used for de novo transcriptome analysis and downstream DEG analysis.

### 3.2. De novo Transcriptome Generation and DEG Analysis for TNRG versus (vs.) TNRC and TNSG vs. TNSC

As the first step for de novo transcriptome analysis, whole transcripts were assembled to be used for the reference transcript by Trinity. A total of 77,072 gene-level contigs were assembled (105,354 contigs were assembled at isoform level). The N50 of the assembled transcript contigs was 1780 base pair (bp), with an average contig length of 1039 bp (Table 1). The genes predicted among the assembled transcript contigs were annotated by a blast search against a protein database. Of these contigs, 32,493 (63,249 isoform contigs) were functionally annotated and uniquely assigned to an NCBI protein accession number by the BlastX database (*E*-value < 0.05; Table S2). The generated whole transcript assembly and its annotation was used as a reference for further DEG analysis. The raw reads and the fasta file of all assembled transcript contigs were submitted at the publicly open database (sequence read archive number: PRJNA764068).

**Figure 1:**
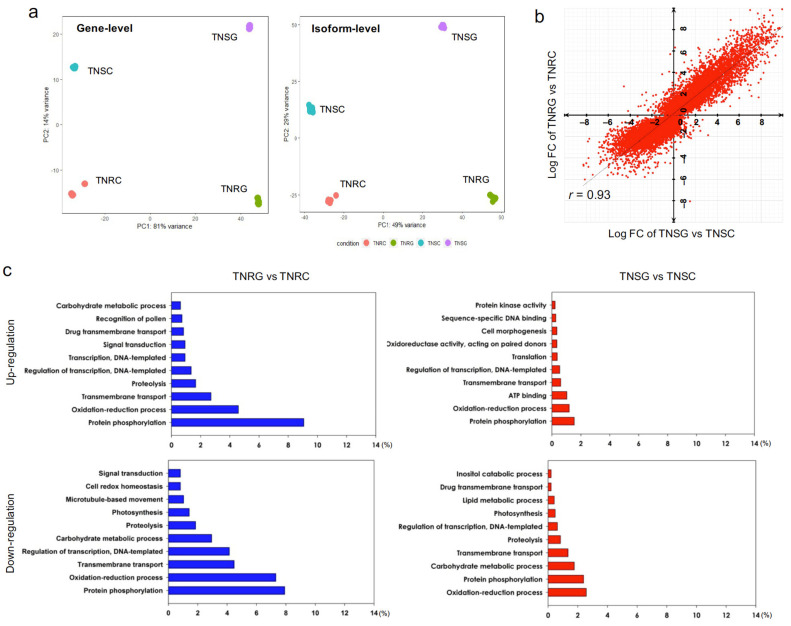
Summary of DEG analysis in TNRG vs. TNRC, and TNSG vs. TNSC. (**a**) Principal coordinates analysis (PCA) of gene-level and isoform-level transcript expression plot of TNS and TNR treated with glyphosate or water. (**b**) Correlation plot of FC variation in TNRG vs. TNRC against TNSG vs. TNSC. Correlation coefficient was determined by the Pearson method. (**c**) Bar graph of the percentage of gene counts of DEGs involved in the biological process category of GO terms.

To conduct the DEG analysis in TNR and TNS with respect to glyphosate treatment, we aligned each group of reads against the whole transcript assembly by the Bowtie2 option in the Trinity software. The aligned reads were quantified to each transcript by RSEM. Real read counts were compiled for four different treatment groups through this process. PCA with four different group alignments showed that most of the variance (91%) among the gene-level read count was on the x-axis (Figure 1a). Glyphosate-treated groups were separated from water-sprayed TNRC and TNSC. Along the y-axis (14% of the variance), the gene expression across the different biotypes of TNR and TNS is clearly separated. The PCA test with the transcript isoform-level read counts also showed similar clustering patterns to that of the gene-level read counts (Figure 1a). This result indicates that many genes were differentially expressed concerning glyphosate treatment and the different biotypes, even within the same genotype. The gene expression of randomly selected DEG from RNA-seq data were validated by RT-qPCR with the contig specific primers (Table S3). For the following analysis, we used gene-level read counts.

Both the TNR and TNS biotypes had many DEGs at 24 h after glyphosate treatment. In the TNR biotype, a total of 23,371 genes were differentially expressed depending upon glyphosate treatment (FDR < 0.05; Table S4); 3640 genes were upregulated (>4 fold); and 3852 genes were downregulated (<−4 fold) in TNRG compared to TNRC. The top 20 of the most differentially expressed genes by glyphosate treatment are listed in Table 2 and Table 3.

In the TNS biotype, a total of 23,663 genes were differentially expressed depending on glyphosate treatment (Table S5). Among them, 3715 genes were upregulated (>4 fold), and 2411 genes were downregulated (<−4) in TNSG (FDR < 0.05). The top 20 of the most differentially expressed genes by glyphosate treatment are listed in Table 4 and Table 5. When we compared the fold change (FC) of each gene in the analysis of TNRG vs. TNRC and TNSG vs. TNSC, the FC value was correlated between each analysis (*r* = 0.93; Figure 1b). Therefore, the glyphosate response of most expressed genes was very consistent between TNR and TNS.

On the basis of the GO term of each protein accession number, we predicted the biological processes of differentially expressed genes. The proportion of gene counts in DEG involved in the same GO term in TNRG vs. TNRC, and TNSG vs. TNSC, was different (Figure 1c). Interestingly, the biological process of transport functions, such as drug transmembrane transport and transmembrane transport, were predominantly up- or downregulated in the TNRG group compared to TNSG, indicating that the transmembrane transport is a typical biological process in TNR after glyphosate treatment. The genes relating to the transcription process were also expressed differentially in TNRG. A larger number of genes relating to signal transduction, protein phosphorylation, and the oxidation-reduction process were down- and upregulated in TNRG. In contrast, the gene relating to protein phosphorylatioin and the oxidation-reduction process was expressed more in TNSG, but the proportion in DEGs was relatively smaller than in TNR.

### 3.3. DEG Groups Dependent on Glyphosate Treatment

**Figure 2:**
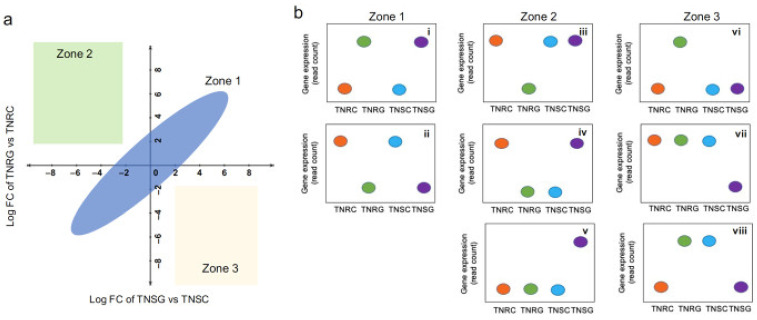
Correlation pattern of DEG in TNRG vs. TNRC, and TNSG vs. TNSC: (**a**) The sorted zone of correlation patterns between TNRG vs. TNRC, and TNSG vs. TNSC. Three zones can be sorted based on FC variation between two DEG analyses; (**b**) Differentially expressed mode summary depending on various read count distributions in TNR and TNS in response to glyphosate.

When we assessed the correlation of DEGs between TNRG vs. TNRC, and TNSG vs. TNSC, the relationships were largely sorted into three zones (Figure 2a). Eight modes of gene count distribution in each treatment were included in the three zones (Figure 2b). The majority of DEGs were sorted in zone 1, with a similar reponse in the TNR and the TNS (Figure 2a). The genes whose read counts were distributed, as shown in the (i) and (ii) modes, were involved in this zone, accompanying similar gene regulation patterns in TNR and TNS after glyphosate treatment (Figure 2b). The genes in these modes may not be specifically regulated in both biotypes by different glyphosate responses. On the other hand, the genes in zone 2 and zone 3 (six types of expression modes) represented the overexpressed genes in TNRG vs. TNRC, but not in TNSG vs. TNSC, and those in TNSG vs. TNSC, but not in TNRG vs. TNRC, repectively. We focused on these groups because these group genes have a higher potential to act specifically on the glyphosate response in glyphosate-resistant plants. In addition, the DEG results in the present study analyzed by edgeR did not include the absolutely expressed genes in the glyphosate-treated samples (0 counts in non-treated samples), but these absolutely expressed genes were included in zones 2 and 3 as well. We could not detect the significant gene showing its read count distribution as (iv) and (viii) that the glyphosate response was reversed in TNR and TNS. In contrast, the present DEG analysis observed the genes in the (iii), (v), (vi), and (vii) modes that should be expressed differentially in different biotypes under glyphosate treatment. To find the dominant functional gene unique to glyphosate treatment, we first analyzed absolutely expressed genes in either biotype by glyphosate treatment, and then collected the genes, including these four modes (iii, v, vi, and vii).

**Figure 3:**
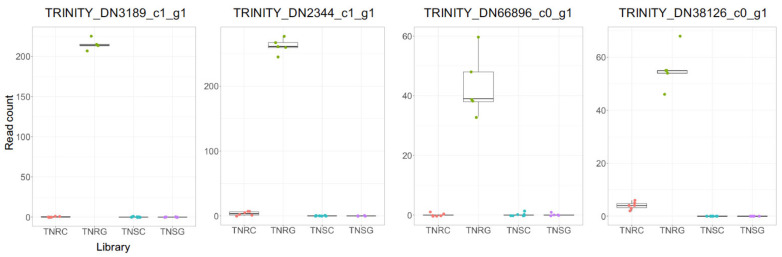
Read count summary of representative genes of upregulated genes in TNR but not in TNS after glyphosate treatment. These genes were selected from the genes listed in Table 6.

A total of 517 genes were upregulated in TNRG (FC > 4), while the corresponding read counts in TNRC, TNSG, and TNSC were near zero (mode vi). However, many transcripts in this mode were expressed with small read counts (less than an average of 20 read counts) so we filtered out these genes. A total of 33 genes remained, of which 15 genes had corresponding protein accession numbers (Table S6). Their read count matrix and protein ids are listed in Table 6. Trinity_DN2344_c1_g1 had the highest read counts in this mode, but its protein function is not identified even though its translated protein had a real open-reading-frame sequence (Table 6). Interestingly, 10 genes in this group were predicted as coding for receptors or transporters on the membrane structure, wherein several were lectin-domain-containing or leucine-rich repeated kinase. The real read count profiles of representative receptor kinases are shown in Figure 3, which is representative of the distribution data as shown in mode vi.

**Figure 4:**
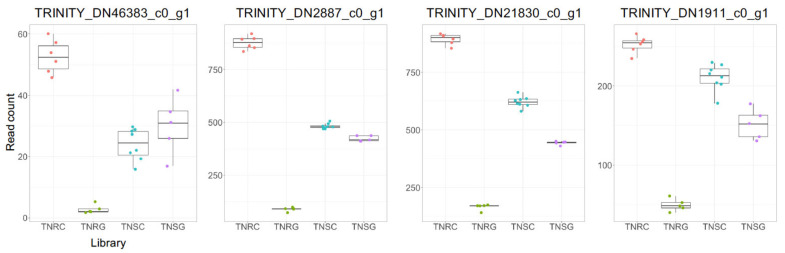
Read count summary of representative genes of downregulated genes in TNR but not in TNS after glyphosate treatment. These genes were selected from the genes listed in Table 7.

Reversely, 26 genes were significantly downregulated in TNRG (FC > 4) but not in TNSG. However, protein id was assigned for only 13 genes (Table 7 and Figure 4). The read count profile of these genes is shown as mode iii, which was sorted in zone 2 (Figure 2b). In this group, we could not find a conserved functional protein family.

We also found that 12 genes were expressed in TNS but not in TNR after glyphosate treatment (mode v in Figure 2b). Six genes among these had a functional protein id (Figure S2). While these genes were coincidentally expressed in TNRG, their read counts were small enough to ignore. We were unable to find absolutely downregulated genes in TNSG (mode vii in Figure 2b). Taken together, absolutely expressed genes existed in both biotypes depending on glyphosate treatment.

### 3.4. DEG Groups between TNR and TNS without Glyphosate Treatment

Even though the majority of the genomic sequences of TNR and TNS are identical, precise sequence variation still exists. The sequence variation, which included the promoter and coding regions, was identified in the whole genome sequencing of another ecotype of *C. canadensis* [23,24]. In a DEG study of the type presented here—between TNR and TNS—it is important to consider the expression of candidate resistance genes under glyphosate-treated and untreated conditions. First, we tried to find differentially expressed genes in TNR and TNS under normal conditions by analyzing the DEGs in TNRC and TNSC. In the results, a total of 10,629 genes were differentially expressed between the two biotypes in normal conditions (FDR < 0.05); 640 genes were expressed higher (FC > 4), and 960 genes were expressed lower (FC < −4) in TNRC compared to TNSC (Tables S6 and S7). When we filtered out low read counts (<20 read average), 127 and 181 genes remained in the up- and downregulated groups, respectively (Tables S7 and S8). Among these, we were able to predict the protein function of 202 genes. Two CYP 450 proteins, an ABC transporter C family member, and seven transmembrane protein kinase genes, including those encoding leucine-rich- and lectin-domain-containing receptor proteins were detected in this list.

**Figure 5:**
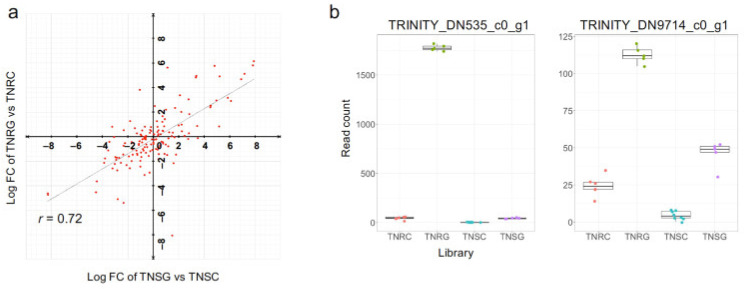
Glyphosate response of DEG in water-sprayed TNR (TNRC) and TNS (TNSC). (**a**) Correlation of FC (log2) in TNSG vs. TNSC, and TNRG vs. TNRC, of DEGs in TNRC vs. TNSC listed in Supplemental Tables S5 and S6. (**b**) Read count summary of two genes whose expressions were higher in TNRC than TNSC, as well as those that were boosted in TNR after glyphosate treatment. These genes were selected from the DEGs listed in Table 8.

To examine the glyphosate response of these 308 differentially expressed genes between TNRC vs. TNSC, we assessed FC in the DEG results from TNRG vs. TNRC, and TNSG vs. TNSC. A total of 140 genes were coincidently expressed in both the DEG analysis results, whose FCs were correlated in TNR and TNS (r = 0.72; Figure 5a). Of these genes, the top 10 of the most differently expressed in TNR are listed in Table 8. TRINITY_DN4487_c0_g1 had the highest expression in TNRC compared to TNSC. However, its gene expression was not altered by glyphosate treatment in both biotypes (Table 8). Interestingly, TRINITY_DN535_c0_gl, a CYP450 protein-encoding gene was originally expressed 5.1 (log2)-fold higher in TNR than TNS. This transcript expression was boosted in TNR after glyphosate treatment, but not in TNS (Table 8 and Figure 5b). TRINITY_DN9714_c0_g1, a COMT-type methyltransferase, was also highly induced in TNR compared to TNS in the glyphosate treatment groups (Figure 5b). Therefore, of the genes originally with higher expression in the TNR compared to TNS, several genes were more highly induced in TNR by glyphosate treatment than in TNS. Even though we observed many genes that were expressed only in TNS, most of them were annotated as uncharacterized genes (Table S7).

### 3.5. Differential Expression of Well-Known NTSR Genes Related to Metabolic Response to Glyphosate

Regarding the well-known *NTSR* candidates, we compiled read counts from the DEG analysis result of the ABC transporters, CYP450, GST, and GTs, which are regarded as important *NTSR* gene families in preventing herbicide damage in weeds.

**Figure 6:**
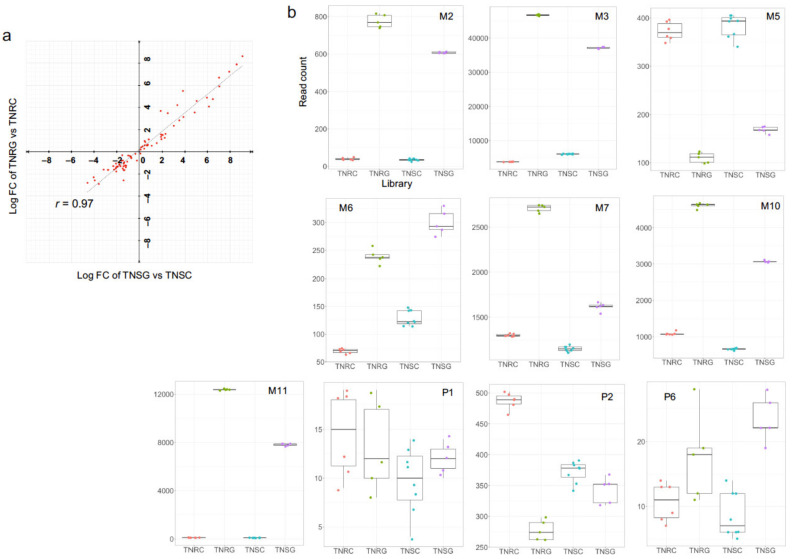
Read counts of representative ABC transporter family genes in TNS and TNR after glyphosate treatment: (**a**) correlation plot of ABC-transporter-assigned transcripts in the DEG analysis of TNRG vs. TNRC, and TNSG vs. TNSC. Note that most ABC transporter gene expression showed similar expression alteration in TNS and TNR after glyphosate treatment; (**b**) Read counts of representative ABC transporter family genes in TNS and TNR after glyphosate treatment. Gene sequences were from previous publication results [21]. Ten transcripts were identified in the present transcriptome.

A total of 134 unique ABC transporter genes (296 at the transcript isoform-level) were assigned in the present TNR and TNS transcriptomes. Of these transcripts, 79 ABC transporter transcripts were differentially expressed in TNR after glyphosate treatment (FDR < 0.05, FC > 4 or <−4; Table S9). In TNS, 90 ABC transporter transcripts were differentially expressed depending on the glyphosate treatment. (FDR < 0.05, FC > 4 or <−4). The FC of ABC transporter gene expression was correlated in both biotypes (r = 0.97, Figure 6a), indicating that most ABC transporters of TNR and TNS were similarly regulated in response to glyphosate.

To compare the previously proposed 17 ABC transporter transcripts with the present transcriptome, each of the reported ABC transporter sequences were aligned independently to the present whole transcript reference [21]. Fifteen transcript sequences were collected from the published data. Of those 15, 12 were identified in our transcriptome (Table S10). However, the M1, M4, and P3 contigs were missing in the present transcriptome. In addition, M3, P4, and P5 were aligned together on a long transcript contig of Trinity DN77_c0_g1 in the whole transcript reference, indicating that these three contigs may be isoforms or fragments of an ABC transporter gene. As identified in Peng and Hereward’s independent studies [13,21], M10 and M11 read counts in TNRG and TNSG were higher than other ABC transporter genes (Figure 6b). In the present study, M3 and M7 transcripts were also upregulated with higher read counts (over 2000 reads) under glyphosate treatment. In contrast, M5 and P2 were downregulated severely in the TNR after glyphosate treatment.

A total of 273 gene contigs were annotated as CYP450 transcripts in the whole transcript reference (455 contigs at transcript isoform-level). Among them, 163 genes were expressed differentially in TNR and TNS after glyphosate treatment (Table S11). The FC of the CYP450 genes in TNRG vs. TNRC was correlated with that of TNSG vs. TNSC (r = 0.95), meaning that most genes in the CYP450 family were regulated similarly in response to glyphosate in TNR and TNS, as observed in ABC transporter gene expression. As we observed in Figure 5b, one CYP450 annotation gene (TRINITY_DN535_c0_gl) was expressed with a higher read count in TNR depending on glyphosate.

Nine GST and 16 GT genes were differentially expressed in both biotypes by glyphosate treatment. The responsive gene number in these families shows a drastic reduction when compared to the previous NTSG target. As previously described, NTSG genes, and the FC and read count distribution of GST and GT genes were similar in both biotypes after glyphosate treatment (Tables S12 and S13).

Therefore, four representative NTSG gene family members were differentially expressed depending on glyphosate, but most FC of their expression did not differ in both TNR and TNS in the present analysis.

## 4. Discussion

Glyphosate is a prominent broad-spectrum herbicide that is important in global agriculture. The evolution of herbicide-resistance in weeds is a growing problem. Therefore, we must gain a better understanding of the evolution of herbicide resistance to inform management decisions. To that end, our study assessed the patterns of DEGs in two different Tennessee biotypes of *C. canadensis* in response to glyphosate treatments. The de novo transcript assembly resulted in the annotation of 32,494 unique transcripts, which is approximately 7000 (28%) more transcripts than previously reported [24]. Third-generation sequencing assisted in a more accurate and complete *C. canadensis* genome reference, allowing us to apply genetic information for herbicide research more effectively [23] and narrow our search for *NTSR* gene candidates.

To date, the research to find glyphosate-resistant genes has focused on the metabolic component genes related to the detoxification of glyphosate. This type of gene is included in the *NTSR* mechanism. Four representative phases are putatively carried out sequentially for glyphosate resistance: activation, conjugation of herbicide, active transport, and degradation of modified herbicide [35]. Each phase is mediated by typical enzyme groups. The present study also focused on the differential gene expression of these enzymes. The CYP 450 family protein was identified as differentially expressed in various herbicide-resistant weeds [36,37,38,39,40]. This protein manages phase I to activate the herbicide for further detoxification in phase II mediated by GST and GT [36]. In glyphosate-resistant morning glory, *Ipomoea purpurea*, the mechanism of glyphosate resistance could be explained by CYP 450 expression, while the expression of the EPSPS gene was unchanged [41]. In the present study, the TRINITY_DN535_c0_gl contig of a CYP 450 gene was naturally expressed higher in wild-type TNR than in TNS (Figure 5b). This gene expression was increased in the glyphosate treatment. According to a recent *C. canadensis* genome annotation, 352 genes were encoded as within the CYP450 family [23]. Our whole transcriptome analysis identified 273 unique CYP 450 transcripts, of which 133 were differentially expressed after glyphosate treatment. In other words, the CYP 450 family appears to play a role in glyphosate resistance in *C. canadensis* (Table S11).

As the mediator enzyme for phase II, both GST and GT were also identified in TNRG and TNSG in the present transcriptome (Tables S12 and S13). However, a small number of transcript species and consistent FCs between glyphosate-treated and non-treated groups of both biotypes were observed. Therefore, these gene families seem to contain glyphosate-responsive genes, but are not regulated differently in different biotypes.

The reduction of glyphosate transport into the cytosol, or the sequestration of activated glyphosate into the vacuole, is the main event of phase III, which is putatively mediated, primarily, by the ABC transporter protein family. In *C. canadensis*, two different studies reported that two ABC transporters play a role in the vacuole sequestration of modified glyphosate, resulting in the reduction of glyphosate toxicity [42,43]. However, we do not know the exact ABC transporter genes that sequester glyphosate. Our present DEG study also showed that many ABC transporter genes were induced with various FCs by glyphosate treatment in TNR and TNS (Figure 6, Table S9). Among the 17 previously reported ABC transporter transcripts in *C. canadensis*, fourteen transcripts were detected in this study [21]. Furthermore, the induction of this gene family after glyphosate treatment was conserved in the transcriptome of *C. bonariensis* [13,14], indicating the possibility that protein function is shared among different weed species.

Interestingly, the M5 and P2 of the ABC transporter transcripts were downregulated in the glyphosate-treated plant leaves (Figure 6b), which indicates that some of the ABC transporter genes are regulated differentially in various routes. Therefore, we carefully select ABC transporter targets to develop experimental glyphosate-resistant transgenic plants in the future.

Many DEGs were identified in the present study that are not among well-known *NTSR* target genes relating to glyphosate response. Most FC among those depending on glyphosate did not differ between TNR and TNS (Figure 1b). Although we found many glyphosate-inducible genes, we hesitate to call them glyphosate-resistance-related genes. Our goal in the present study is to identify glyphosate-resistance related genes by isolating glyphosate-responding genes in the glyphosate-resistant weed transcriptome. Initially, the genetic variation between the TNR and TNS biotypes was fewer sequence variants than the other biotypes in the previous study [24]. However, even small genetic variations can affect an important genetic trait, which was verified in the present study. Through the present study, we carefully suggested genes sorted in zone II and zone III as potential glyphosate-resistant candidates (Figure 2a,b), among which gene expression was differentially distributed in different biotypes depending on glyphosate treatment. (Figure 3 and Figure 4, and Figure S2). Of note, 140 genes that had higher expression in TNR under normal conditions has increased expression in glyphosate-treated plants (Tables S7 and S8). In addition, the present study proposed 33 previously unidentified candidate genes that were specifically and sensitively induced only in the TNR biotype under glyphosate treatment (Table 6 and Table S6, Figure 3). Of these genes, those encoding membrane-bound receptor kinase proteins were typically upregulated in TNR after glyphosate treatment, but not in TNS (Figure 3). These proteins generally mediate the transfer of extracellular signals into the cytoplasm. Lectin receptor-like kinase (LecRLK) is the typical plant kinase protein localized on the plasma membrane in many crop plants [44,45,46]. Because of the extracellular domain variety of LecRLKs, these family members perceive various environmental cues, microbial stimuli, and plant growth/development regulators. Until now, any cellular membrane components in signaling herbicide uptake or detoxification have not been reported. An interesting insight relating to this result was recently reported: glyphosate affected jasmonic acid (JA) levels and green leaf volatiles (GLV) by lipid peroxidation [47]. Although the mechanism whereby the biosynthesis or perception of JA and GLVs are affected by glyphosate is mostly unknown, the plant damage caused by glyphosate may cause a phytohormone response downstream. The signal receptor or receptor kinase probably participated in this process. For instance, the *Nicotiana* lectin receptor kinase 1 has been found to suppress salicylic acid caused by insect attack, thereby inducing a JA-mediated-plant defense mechanism [48,49]. If glyphosate causes damage similar to an insect attack, JA may be a phytohormone linker to be perceived as a signal from lectin receptor kinase.

## 5. Conclusions

Recently, several glyphosate-resistant genes that play a role in metabolic resistance have been proposed for use in transgenic plants [13,14,21,39]. A good candidate gene of the ABCC-type transported from glyphosate-resistant grass (*Echinochloa colona*; EcABCC8), evolved in a Western Australia agricultural field, was used to genetically engineer crops for glyphosate resistance. Transgenic rice, maize, and soybean overexpressing an EcABCC8 ortholog resulted in endowing glyphosate resistance, indicating that the identical glyphosate-resistant trait could be expected in different crop plants with the grass ABCC8 gene [50]. The present DEG study suggested 240 annotated genes that were expressed higher only in either the TNR or the TNS of *C. canadensis* after glyphosate treatment, and originally expressed in the TNR. In addition, we found a CYP 450 and several membrane-bound protein kinases expressed specifically in the glyphosate-resistant biotype. These genes could be utilized for the practical agricultural purpose of overcoming the emergence of glyphosate-resistant weeds.

## Figures and Tables

**Table 1:** Statistics of Illumina sequencing and de novo transcriptome analysis in *C. canadensis*. The de novo transcriptome was assembled from a total of 24 libraries of TNR and TNS biotypes, with and without glyphosate treatment.

Description	De novo Assembly Statistics
Total assembled reads	100,204,924
Total isoform level contigs	105,354
Total gene level contigs	77,072
Contig N50 (bp)	1780
Average contig length (bp)	1039.7
GC (%)	38.21

**Table 2:** The gene list of the top 20 upregulated genes in TNRG vs. TNRC. De novo transcriptome analysis was performed by Trinity with 11 libraries (6 glyphosate-treated and 5 non-treated leaves). DEGs were analyzed by edgeR software. Statistically, a total of 3640 transcripts were upregulated over 4 FC in TNRG than TNRC (FDR < 0.05). Among them, the top 20 genes are listed in this table.

Contig ID	LogFC	FDR	Protein Accession ID	Description
TRINITY_DN905_c0_g1	11.8	0	XP_021979261.1	Uncharacterized protein LOC110875373
TRINITY_DN13727_c0_g1	10.3	4.0 × 10^−249^	OTG03028.1	F-box domain-containing protein
TRINITY_DN27339_c0_g1	10.2	0	XP_023758053.1	Glutathione S-transferase parA
TRINITY_DN36883_c0_g1	10.3	0	PWA57492.1	Aminotransferase, class I/classII
TRINITY_DN1722_c0_g2	10.1	0	PWA51732.1	Hypothetical protein CTI12_AA461460
TRINITY_DN66403_c0_g1	10	0	XP_024958679.1	Uncharacterized protein LOC112499602
TRINITY_DN6438_c0_g1	9.9	0	XP_024988661.1	AAA-ATPase At3g50940-like
TRINITY_DN4388_c0_g1	9.9	0	XP_022010209.1	Uncharacterized protein LOC110909706
TRINITY_DN11441_c0_g1	9.7	1.2 × 10^−165^	XP_022008773.1	Uncharacterized protein LOC110908182
TRINITY_DN3079_c0_g1	9.7	0	PWA86457.1	NADH-ubiquinone reductase complex 1 MLRQ subunit
TRINITY_DN37237_c0_g1	9.6	0	PLY74787.1	Hypothetical protein LSAT_6X72041
TRINITY_DN56890_c0_g1	9.4	1.3 × 10^−136^	PWA59237.1	Hypothetical protein CTI12_AA394960
TRINITY_DN2953_c0_g1	9.4	1.5 × 10^−131^	XP_022005344.1	Inactive ATP-dependent zinc metalloprotease FTSHI 3
TRINITY_DN11270_c0_g1	9.3	1.4 × 10^−128^	XP_022000952.1	Ultraviolet-B receptor UVR8-like
TRINITY_DN5461_c0_g1	9.3	2.3 × 10^−294^	XP_021999019.1	Phospholipase A1-Igamma2, chloroplastic-like
TRINITY_DN68599_c0_g1	9.3	1.1 × 10^−122^	PLY95867.1	Hypothetical protein LSAT_5X173860
TRINITY_DN282_c0_g1	9.3	0	AAT72931.1	Cascarilladiene synthase
TRINITY_DN48830_c0_g1	9.2	2.9 × 10^−112^	XP_023756578.1	Flavin-containing monooxygenase 1
TRINITY_DN669_c1_g1	9.2	0	AAT72931.1	Cascarilladiene synthase
TRINITY_DN310_c0_g3	9.1	1.0 × 10^−248^	PWA98523.1	ABC transporter Tap-like

**Table 3:** The gene list of the top 20 downregulated genes in TNRG vs. TNRC. De novo transcriptome analysis followed by DEG analysis were performed by Trinity and edgeR softwaren with the same cDNA libraries used in Table 2. A total of 3852 genes were downregulated significantly over −4 FC in TNRG than TNRC (FDR < 0.05). This table lists the top 20 genes of the downregulated genes.

Contig ID	LogFC	FDR	Protein Accession ID	Description
TRINITY_DN3992_c0_g1	−8.3	0	XP_021976551.1	Chlorophyll a-b binding protein of LHCII type 1
TRINITY_DN2551_c0_g1	−8.3	0	WP_124736833.1	Hypothetical protein
TRINITY_DN9403_c0_g1	−8.1	0	PWA49348.1	Chlorophyll A-B binding protein
TRINITY_DN11212_c0_g1	−8.0	2.0 × 10^−48^	XP_023760034.1	Uncharacterized protein LOC111908441
TRINITY_DN14399_c0_g1	−7.9	3.9 × 10^−45^	XP_023762447.1	WD repeat and HMG-box DNA-binding protein 1
TRINITY_DN42_c2_g1	−7.8	0	KVI00865.1	Chlorophyll A-B binding protein
TRINITY_DN18783_c0_g1	−7.8	7.2 × 10^−43^	XP_019249685.1	Uncharacterized protein LOC109228892
TRINITY_DN38340_c0_g1	−7.7	8.6 × 10^−41^	XP_021973416.1	Origin of replication complex subunit 1A-like
TRINITY_DN42_c4_g1	−7.4	0	XP_023739911.1	Chlorophyll a-b binding protein of LHCII type 1
TRINITY_DN14573_c0_g1	−6.9	5.0 × 10^−22^	XP_016071745.1	Uncharacterized protein LOC107539687
TRINITY_DN14043_c0_g1	−6.8	5.3 × 10^−53^	XP_023763882.1	High mobility group B protein 7
TRINITY_DN2800_c0_g1	−6.8	2.1 × 10^−114^	XP_023761206.1	Palmitoyl-monogalactosyldiacylglycerol delta-7 desaturase
TRINITY_DN1183_c1_g1	−6.8	2.1 × 10^−51^	XP_024991237.1	Zinc finger protein At1g68190
TRINITY_DN200_c0_g2	−6.7	0	AEY78525.1	Chlorophyll a/b-binding protein
TRINITY_DN1119_c0_g1	−6.7	0	KVI00865.1	Chlorophyll A-B binding protein
TRINITY_DN70454_c0_g1	−6.7	1.5 × 10^−19^	XP_022016163.1	Uncharacterized protein At1g04910
TRINITY_DN63729_c0_g1	−6.5	5.9 × 10^−17^	PWA63735.1	Phosphate-induced protein 1
TRINITY_DN15048_c0_g1	−6.4	3.8 × 10^−16^	XP_022037435.1	Pathogenesis-related protein PR-1-like
TRINITY_DN57166_c0_g1	−6.4	3.6 × 10^−39^	PWA93616.1	Hypothetical protein CTI12_AA069020
TRINITY_DN56621_c0_g1	−6.4	2.1 × 10^−15^	XP_022008540.1	Uncharacterized protein LOC110907935

**Table 4:** The gene list of the top 20 upregulated genes in TNSG vs. TNSC. De novo transcriptome analysis was performed by Trinity with 13 libraires (5 glyphosate-treated and 8 non-treated leaves). EdgeR was used for DEG analysis. A total of 3715 genes were upregulated (FC > 4) in TNSG than TNSC (FDR < 0.05). The top 20 genes are listed in this table.

Contig ID	LogFC	FDR	Protein Accession ID	Description
TRINITY_DN65437_c0_g1	10.4	0	XP_021965748.1	Protein mesh
TRINITY_DN1160_c1_g1	10.2	0	XP_026462482.1	Vitellogenin-A1-like
TRINITY_DN4388_c0_g1	9.9	0	XP_022010209.1	Uncharacterized protein LOC110909706
TRINITY_DN1722_c0_g2	9.9	0	XP_021975061.1	Uncharacterized protein LOC110870166
TRINITY_DN1160_c0_g1	9.8	0	XP_026462481.1	Vitellogenin-A1-like
TRINITY_DN56688_c0_g1	9.8	0	PWA98881.1	Alternative oxidase 1D
TRINITY_DN1160_c2_g1	9.7	2.1 × 10^−201^	XP_017781017.1	Vitellogenin-A1-like
TRINITY_DN2458_c0_g1	9.7	6.7 × 10^−206^	AAH21837.1	Ubc protein
TRINITY_DN905_c0_g1	9.7	0	XP_021979261.1	Uncharacterized protein LOC110875373
TRINITY_DN14914_c0_g1	9.5	7.2 × 10^−167^	XP_001654240.1	Maternal protein exuperantia-2
TRINITY_DN745_c0_g1	9.3	0	OTG36297.1	putative heat shock protein 70 family
TRINITY_DN6494_c0_g1	9.3	4.7 × 10^−281^	XP_026272532.1	Vitellogenin-1-like
TRINITY_DN16886_c0_g1	9.3	2.8 × 10^−147^	ACT80192.1	Cytochrome c oxidase subunit 1 (mitochondrion)
TRINITY_DN5166_c0_g1	9.2	0	ABV60316.1	Putative ADP/ATP translocase
TRINITY_DN10008_c0_g1	9.1	6.2 × 10^−265^	PWA80350.1	Hypothetical protein CTI12_AA197740
TRINITY_DN28371_c0_g1	9.1	2.6 × 10^−131^	XP_002066234.1	Elongation factor 1-α 1
TRINITY_DN1550_c0_g1	9.0	2.8 × 10^−225^	AID52928.1	60S acidic ribosomal protein P0
TRINITY_DN19097_c0_g1	9.0	1.5 × 10^−117^	AXX71242.1	Juvenile hormone
TRINITY_DN3463_c0_g1	8.8	0	XP_024984476.1	ADP, ATP carrier protein, mitochondrial
TRINITY_DN16233_c0_g1	8.7	4.0 × 10^−105^	YP_009487737.1	Cytochrome c oxidase subunit III (mitochondrion)

**Table 5:** The top 20 gene list of downregulated genes in TNSG vs. TNSC. De novo transcriptome and DEG analyses were performed by Trinity and edgeR with the same libraries as Table 4. A total of 2411 genes were significantly downregulated by glyphosate treatment in TNS (FDR < 0.05). The top 20 downregulated genes are listed.

Contig ID	LogFC	FDR	Protein Accession ID	Description
TRINITY_DN28174_c0_g1	−8.1	2.0 × 10^−19^	XP_024970575.1	Septum-site-determining protein minD homolog, chloroplastic
TRINITY_DN9403_c0_g1	−6.0	0	PWA49348.1	Chlorophyll A-B binding protein
TRINITY_DN17513_c0_g1	−5.7	5.4 × 10^−10^	PWA54703.1	Solute carrier family 25 member 44
TRINITY_DN17284_c0_g1	−5.6	9.4 × 10^−10^	XP_023758730.1	Uncharacterized protein LOC111907163
TRINITY_DN20553_c0_g1	−5.6	7.9 × 10^−25^	PWA85753.1	Protochlorophyllide oxidoreductase
TRINITY_DN42_c4_g1	−5.6	0	XP_023739911.1	Chlorophyll a-b binding protein of LHCII type 1
TRINITY_DN57253_c0_g1	−5.5	2.4 × 10^−9^	PWA80339.1	Coiled-coil domain containing protein 109
TRINITY_DN58008_c0_g1	−5.5	2.1 × 10^−9^	XP_022009704.1	β-galactosidase 3
TRINITY_DN37787_c0_g1	−5.4	4.8 × 10^−21^	XP_023765231.1	Basic 7S globulin 2-like isoform X2
TRINITY_DN67280_c0_g1	−5.4	2.0 × 10^−288^	PWA87944.1	Hypothetical protein CTI12_AA124470
TRINITY_DN51763_c0_g1	−5.3	5.5 × 10^−8^	PWA59983.1	DNA-binding domain-containing protein
TRINITY_DN274_c0_g1	−5.3	0	PWA65034.1	Peptidase T2, asparaginase 2, nucleophile aminohydrolase
TRINITY_DN57640_c0_g1	−5.3	1.2 × 10^−7^	PWA39470.1	Protochlorophyllide oxidoreductase
TRINITY_DN58011_c0_g1	−5.2	9.2 × 10^−8^	XP_021990878.1	Uncharacterized protein LOC110887609
TRINITY_DN18290_c0_g1	−5.1	3.1 × 10^−7^	OMO80329.1	Short-chain dehydrogenase/reductase SDR
TRINITY_DN8861_c0_g1	−5.1	1.0 × 10^−97^	XP_021986658.1	Short-chain dehydrogenase TIC 32
TRINITY_DN30954_c0_g1	−5.1	1.5 × 10^−6^	XP_023753002.1	Peroxidase 19-like
TRINITY_DN24284_c0_g1	−5.0	1.6 × 10^−6^	OTG12714.1	Putative serine/threonine/dual specificity protein kinase
TRINITY_DN43940_c0_g1	−4.9	2.6 × 10^−6^	PLY91593.1	Hypothetical protein LSAT_7X10301
TRINITY_DN48832_c0_g1	−4.9	4.2 × 10^−6^	PWA97992.1	Cytochrome P450

**Table 6:** Gene list of upregulated genes only in TNRG. These genes were expressed in TNR but not in TNS after glyphosate treatment (FDR < 0.05). Thirty-three genes were filtered by removing the gene expressed with small read counts (<20) from 517 upregulated genes only in TNR. Of these genes, 15 genes had corresponding protein accession numbers in Blast X. This table lists these 15 genes (n.a. = not available).

Contig ID	TNRG vs. TNRC		TNSG vs. TNSC		Protein Accession ID	Description
	Log FC	FDR	LogFC	FDR		
TRINITY_DN3189_c1_g1	8.9	0	n.a.	n.a.	XP_023760056.1	Uncharacterized protein LOC111908459
TRINITY_DN66896_c0_g1	8.4	4.9 × 10^−68^	n.a.	n.a.	XP_022012333.1	Wall-associated receptor kinase-like 8
TRINITY_DN51395_c0_g1	7.3	3.7 × 10^−33^	n.a.	n.a.	PWA42696.1	Homeodomain-like protein
TRINITY_DN17396_c0_g1	6.9	1.1 × 10^−55^	n.a.	n.a.	XP_021968739.1	Plasmamembrane calcium-transporting ATPase 12
TRINITY_DN67025_c0_g1	6.6	1.3 × 10^−46^	n.a.	n.a.	KVI07684.1	Leucine rich repeat 4
TRINITY_DN2344_c1_g1	6.1	0	n.a.	n.a.	XP_023771967.1	Kinesin light chain 3 isoform X2
TRINITY_DN65261_c0_g1	5.7	3.9 × 10^−30^	n.a.	n.a.	XP_023769670.1	NADH dehydrogenase iron-sulfur protein 8
TRINITY_DN17899_c0_g1	5.6	4.2 × 10^−66^	n.a.	n.a.	PWA34905.1	B-type lectin domain-containing protein
TRINITY_DN67848_c0_g1	4.7	2.3 × 10^−51^	n.a.	n.a.	OTF95985.1	Malectin-binding domain-containing protein
TRINITY_DN15755_c0_g1	3.9	5.6 × 10^−27^	n.a.	n.a.	PWA66199.1	Toll/interleukin-1 receptor (TIR) domain protein
TRINITY_DN7012_c0_g1	3.8	4.5 × 10^−24^	n.a.	n.a.	PWA81859.1	Toll/interleukin-1 receptor (TIR) domain protein
TRINITY_DN38126_c0_g1	3.7	1.6 × 10^−60^	n.a.	n.a.	XP_022016073.1	L-type lectin-domain containing receptor kinase
TRINITY_DN48964_c0_g1	3.5	7.7 × 10^−53^	n.a.	n.a.	XP_022011897.1	L-type lectin-domain containing receptor kinase
TRINITY_DN33216_c0_g1	3.2	7.7 × 10^−41^	n.a.	n.a.	XP_022012440.1	L-type lectin-domain containing receptor kinase
TRINITY_DN15265_c0_g2	2.2	3.3 × 10^−16^	−0.2	0.8	PWA39743.1	Copper centre Cu(A)

**Table 7:** Gene list of downregulated genes only in TNRG. These genes were downregulated in TNR but not in TNS after glyphosate treatment (FDR < 0.05). Twenty-six genes were filtered by removing the gene expressed with small read counts (<20) from 97 downregulated genes only in TNR. Of these genes, 13 genes had corresponding protein accession numbers, which are listed in this table.

Contig ID	TNRG vs. TNRC		TNSG vs. TNSC		Protein Accession ID	Description
	Log FC	FDR	LogFC	FDR		
TRINITY_DN46383_c0_g1	−4.2	5.9 × 10^−60^	0.4	2.2 × 10^−2^	XP_022011882.1	Uncharacterized protein LOC110911561
TRINITY_DN2887_c0_g1	−3.3	0	−0.1	1.2 × 10^−3^	XP_022002942.1	β-glucosidase 24-like
TRINITY_DN14379_c0_g1	−2.7	2.8 × 10^−52^	−0.5	2.4 × 10^−5^	XP_023732528.1	GRAVITROPIC IN THE LIGHT 1-like isoform X1
TRINITY_DN22567_c0_g1	−2.6	4.2 × 10^−60^	−0.5	2.0 × 10^−7^	PWA81344.1	Hypothetical protein CTI12_AA064670
TRINITY_DN14266_c0_g1	−2.5	6.6 × 10^−30^	−0.3	4.6 × 10^−2^	XP_021982692.1	Cysteine-rich repeat secretory protein 60-like
TRINITY_DN3134_c0_g1	−2.5	1.2 × 10^−86^	−0.1	4.7 × 10^−1^	XP_021969889.1	Isoflavone reductase homolog
TRINITY_DN21830_c0_g1	−2.4	0	−0.4	2.2 × 10^−31^	XP_024983361.1	Uncharacterized protein LOC112519462
TRINITY_DN36753_c0_g1	−2.4	6.7 × 10^−70^	0.5	5.3 × 10^−10^	XP_010273654.1	Mannose/glucose-specific lectin-like
TRINITY_DN14143_c0_g1	−2.4	4.0 × 10^−23^	−0.4	2.2 × 10^−2^	PWA85337.1	Hypothetical protein CTI12_AA151440
TRINITY_DN1911_c0_g1	−2.3	6.1 × 10^−169^	−0.4	1.7 × 10^−10^	XP_023773027.1	Sulfate transporter 3.1-like
TRINITY_DN9813_c0_g1	−2.3	1.9 × 10^−32^	0.4	7.9 × 10^−3^	XP_024959450.1	Protein LNK1-like isoform X1
TRINITY_DN16564_c0_g1	−2.3	1.4 × 10^−31^	−0.2	1.2 × 10^−1^	PWA68294.1	WRKY domain-containing protein
TRINITY_DN65314_c0_g1	−2.1	1.5 × 10^−38^	−0.4	7.8 × 10^−3^	XP_023763206.1	Blue copper protein

**Table 8:** Top 10 gene list of significantly up- or downregulated genes in TNRC vs. TNSC. The genes whose expressions were up- or downregulated in TNR compared to TNS without glyphosate treatment are listed in this table (FC > 4 or FC < −4; FDR < 0.05). When we filtered out low read counts (<20 read average), 127 and 181 genes remained in the up- and downregulated groups, respectively (Tables S5 and S6). Among these, we were able to predict the protein function of 202 genes. The top 10 genes having a higher or lower FC with protein accession numbers are listed in this table.

Contig ID		TNRC vs. TNSC		TNRG vs. TNRC		TNSG vs. TNSC		Protein Accession ID	Description
		LogFC	FDR	LogFC	FDR	LogFC	FDR		
Up-regulated in TNR	TRINITY_DN4887_c0_g1	7.6	0	0.7	2.9 × 10^−65^	0.5	5.5 × 10^−1^	XP_023740558.1	Protein LURP-1-related 10-like
	TRINITY_DN535_c0_g1	5.1	6.6 × 10^−83^	5.2	0	4.9	2.2 × 10^−73^	PWA57088.1	Cytochrome P450
	TRINITY_DN5825_c3_g2	4.8	0	0.3	1.5 × 10^−28^	0.8	1.2 × 10^−6^	PWA99700.1	Armadillo-type fold
	TRINITY_DN5327_c0_g1	4.2	8.2 × 10^−57^	1.1	1.0 × 10^−18^	5.6	1.1 × 10^−180^	KVH87673.1	Ankyrin repeat-containing protein
	TRINITY_DN15014_c0_g1	4.2	2.8 × 10^−89^	0.4	0	0.2	8.5 × 10^−1^	XP_023756443.1	Uncharacterized protein LOC111904992
	TRINITY_DN12399_c0_g1	3.7	3.0 × 10^−39^	0.7	1.6 × 10^−5^	1.2	1.6 × 10^−2^	PWA74669.1	Glutamyl-tRNA(Gln) amidotransferase
	TRINITY_DN3064_c0_g2	3.4	9.3 × 10^−53^	0.2	0.147264	−0.2	6.6 × 10^−1^	OWM86275.1	Hypothetical protein CDL15_Pgr011099
	TRINITY_DN4900_c1_g1	2.8	1.9 × 10^−60^	0.9	1.3 × 10^−16^	0.8	4.8 × 10^−4^	PLY78555.1	Hypothetical protein LSAT_1X84440
	TRINITY_DN9714_c0_g1	2.6	4.5 × 10^−26^	2.1	3.0 × 10^−66^	3.4	1.6 × 10^−54^	PWA81921.1	O-methyltransferase, COMT-type
	TRINITY_DN830_c0_g2	2.3	0	1.5	0	0.3	4.0 × 10^−160^	RCW19059.1	Hypothetical protein GLYMA_13G018000
Up-regulated in TNS	TRINITY_DN3422_c0_g1	−9.5	0	n.a.	n.a.	−0.5	5.4 × 10^−16^	PWA34949.1	Ribonuclease H-like domain-containing protein
	TRINITY_DN16145_c0_g1	−9.5	5.3 × 10^−151^	n.a.	n.a.	−0.1	5.9 × 10^−1^	PWA56637.1	RNA-directed DNA polymerase
	TRINITY_DN10659_c0_g1	−9.5	2.2 × 10^−159^	n.a.	n.a.	−0.2	1.2 × 10^−2^	XP_018725631.1	CHROMATIN REMODELING 24
	TRINITY_DN10231_c0_g1	−9.3	0	n.a.	n.a.	−2.1	4.6 × 10^−115^	XP_023748705.1	Translocase of chloroplast 34, chloroplastic
	TRINITY_DN6562_c0_g1	−9.1	1.0 × 10^−276^	n.a.	n.a.	−0.6	9.2 × 10^−15^	XP_021985853.1	Uncharacterized protein LOC110882061
	TRINITY_DN1776_c0_g1	−8.8	0	n.a.	n.a.	−0.1	8.1 × 10^−2^	PLY61944.1	Hypothetical protein LSAT_5X69860
	TRINITY_DN11878_c0_g1	−8.7	5.9 × 10^−219^	n.a.	n.a.	0.9	1.1 × 10^−45^	XP_024984807.1	FACT complex subunit SPT16 isoform X1
	TRINITY_DN65613_c0_g1	−8.7	2.2 × 10^−211^	n.a.	n.a.	−0.7	4.6 × 10^−14^	XP_021992293.1	Uncharacterized protein LOC110889094
	TRINITY_DN9729_c0_g1	−8.6	4.1 × 10^−217^	n.a.	n.a.	0.2	2.0 × 10^−2^	OTF97831.1	Mitogen-activated protein (MAP) kinase kinase
	TRINITY_DN8641_c0_g1	−8.6	1.4 × 10^−87^	n.a.	n.a.	0.6	2.3 × 10^−9^	XP_022025290.1	DNA-directed RNA polymerase

## Data Availability

The data presented in this study will be available on the SRA database (http://www.ncbi.nlm.nih.gov/sra) accessed on 1 October 2021 (Accession ID: PRJNA764068).

## Supplementary Materials

The following are available online at https://www.mdpi.com/article/10.3390/genes12101616/s1: Figure S1. Experimental flowchart for glyphosate treatment and DEG analysis. The leaf tissues were collected from TNRG and TNRC; Figure S2. Upregulated genes only in TNS but not in TNR after glyphosate treatment. (a) Gene list of upregulated genes by glyphosate treatment only in TNS. (b) Representative read count summary of listed genes in panel (a); Table S1. Primers of the randomly selected genes from RNA-seq data for validation of RNA-seq analysis by RT-qPCR; Table S2. Whole assembled transcript list. All transcripts assigned to NCBI nr protein identity are listed here. A total of 63,249 isoform contigs were assembled, which have 32,943 unique protein identities; Table S3. Validation of differential expressed genes of TNRG vs. TNRC, and TNSG vs. TNSC in RNA-seq analysis with RT-qPCR; Table S4. The entire list of differentially expressed genes in glyphosate-treated TNR compared to water-sprayed (FDR < 0.05); Table S5. The entire list of differentially expressed genes in glyphosate-treated TNS compared to water-sprayed (FDR < 0.05); Table S6. The entire list of DEGs only in TNR, not in TNS, after glyphosate treatment (FDR < 0.05); Table S7. The entire list of upregulated genes in TNR under normal conditions. The DEG FC in TNRG vs. TNRC, and TNSG vs. TNSC are also listed together (FDR < 0.05); Table S8. The entire list of downregulated genes in TNR under normal condtions. The DEG FC in TNRG vs. TNRC, and TNSG vs. TNSC were also listed together (FDR < 0.05); Table S9. The summary of FC and read count of ABC transporter gene in TNR and TNS after glyphosate treatment. (FDR < 0.05); Table S10. The ABC transporter gene list in the present transcriptome based on previously proposed ABC tranporter genes by Peng et al. [21] Twelve previously reported transcripts were aligned to 10 transcripts in the whole transcriptome. Especially, the TRINITY_DN77 transcript was aligned with M3, P4, and P5, which were predicted as separate ABC genes before; Table S11. The summary of FC and read counts of the CYP450 assigned gene in TNR and TNS after glyphosate (FDR < 0.05); Table S12. The summary of FC and the read counts of GST in TNR and TNS after glyphosate treatment (FDR < 0.05); Table S13. The summary of FC and read counts of GT in TNR and TNS after glyphosate treatment (FDR < 0.05).
