# Supplementary material for: Novel Candidate Genes Differentially Expressed in Glyphosate-Treated Horseweed (Conyza canadensis)
Source: Genes (Basel). 2021 Oct 14;12(10):1616. doi: 10.3390/genes12101616 (PMC8535903; doi:10.3390/genes12101616)

a

Contig ID	TNRG vs TNRC		TNSG vs TNSC		Protein accession no.	Description
	log FC	FDR	log FC	FDR		
TRINITY_DN27451_c0_g1	3.3	2.3E-09	2.4	7E-30	PWA94409.1	Hypothetical protein CTI12_AA062430
TRINITY_DN6162_c0_g1	3.3	4.2E-04	3.0	8E-37	PWA81817.1	TMV resistance protein N
TRINITY_DN30253_c0_g1	4.5	3.3E-10	3.8	2E-46	XP_023764785.1	Protein ALP1-like
TRINITY_DN4356_c0_g2	4.9	2.4E-13	4.0	2E-38	XP_022012719.1	Wall-associated receptor kinase-like 2 isoform X2
TRINITY_DN20700_c0_g1	2.3	3.4E-06	2.6	3E-24	XP_024991351.1	Phosphoenolpyruvate carboxylase kinase 2-like
TRINITY_DN25233_c0_g1	2.6	1.5E-04	3.1	7E-28	PWA85832.1	Hypothetical protein CTI12_AA146110

b

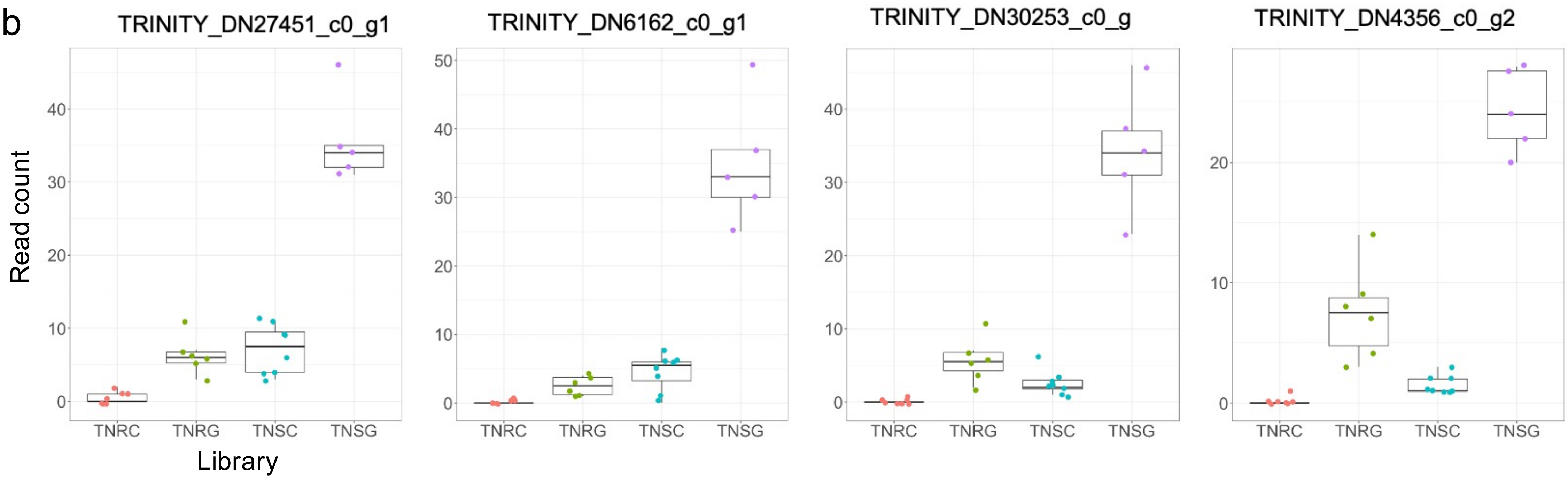

Supplement: Supplementary file 1 [file genes-12-01616-s001.zip › genes-1360991-supplementary/Figure S2.pdf]
