# Supplementary material for: Novel Candidate Genes Differentially Expressed in Glyphosate-Treated Horseweed (Conyza canadensis)
Source: Genes (Basel). 2021 Oct 14;12(10):1616. doi: 10.3390/genes12101616 (PMC8535903; doi:10.3390/genes12101616)
Supplement: Supplementary file 1 [file genes-12-01616-s001.zip › genes-1360991-supplementary/Table S1.pdf]

Table S1. Primers of the randomly selected genes from RNA-seq data for validation of RNA-seq analysis by RT-qPCR.

Contig ID	Orientation	Sequence (5’-3’)
TRINITY_DN310_c0_g1	Forward	GGAGTGTGGGACAAAGGCAACTTC
	Reverse	TGCATACCCTGCATCAATGACTAAAACC
TRINITY_DN4460_c0_g1	Forward	TGACCGTTCTCAAGCACAAGAATTCG
	Reverse	GGTCTCAGAGGGAGGGGAGAAT
TRINITY_DN1752_c0	Forward	GTGCGGGAAGAGCTCTGTAATCG
	Reverse	GGTGGCGGCTTCTATGATCTCG
TRINITY_DN11995_c0_g1	Forward	CGAGTCTGCTCAGGGAAAACGAC
	Reverse	CGAACCTGAACCCCCTGATACC
TRINITY_DN9863_c0_g	Forward	CAAGGATCTTTTGATGGCAGGAACAGC
	Reverse	GTCAGCTGAACTCCTGGTAGGC
TRINITY_DN17441_c0_g1	Forward	GCCGCGATATCTGAGGGATTACG
	Reverse	CCATCGTTCGGGTCTAAAAACACGAC
TRINITY_DN672_c0_g1	Forward	GAGCGGATCCCAGAACGTATTGTTC
	Reverse	GTCTCTGATTGTTTCAGGGCTTCC
TRINITY_DN7231_c0_g1	Forward	GGAGAGCATCACCGTGGTTATGTTG
	Reverse	CCTGGTTGCATGGATTCCCAGAAG
Tubulin	Forward	AGCAATAGTGCATGGGGTTCTCG
	Reverse	CTCACGTTTAGCAGCTCTGCTTTG
