# Supplementary material for: Novel Candidate Genes Differentially Expressed in Glyphosate-Treated Horseweed (Conyza canadensis)
Source: Genes (Basel). 2021 Oct 14;12(10):1616. doi: 10.3390/genes12101616 (PMC8535903; doi:10.3390/genes12101616)
Supplement: Supplementary file 1 [file genes-12-01616-s001.zip › genes-1360991-supplementary/Table S3.pdf]

Table S3. Validation of differential expressed genes of TNRG vs TNRC and TNSG vs TNSC in RNA-seq analysis with RT-qPCR. Tubulin gene was used as an internal control for normalization for RT-qPCR data analysis. The ratio of relative gene expression (R.E.) was determined by $2^{-\Delta\Delta Ct}$ equation. For RT-qPCR, three technical replicates of three glyphosate- or water sprayed horseweed leaves were used for analysis (* p-value <0.05 , ** p-value <0.01). The significance of F.C. of RNA-seq indicated FDR (** FDR < 0.01).

Contig ID	TNRG vs TNRC		TNSG vs TNSC	
	FC of RNA-seq (log2)	RE of RT-qPCR(log2)	FC of RNA-seq(log2)	RE of RT-qPCR(log2)
TRINITY_DN310_c0_g2	4.3**	5.2**	4.2**	3.5**
TRINITY_DN4460_c0_g1	1.9**	1.1*	1.4**	1.7**
TRINITY_DN9863_c0_g1	1.2**	1.3*	1.1**	1.7*
TRINITY_DN7231_c0_g1	-0.7**	1.0	-1.1**	0.4
TRINITY_DN1752_c0_g1	-1.2**	-0.2	-1.1**	0.6
TRINITY_DN17441_c0_g1	-1.7**	-2.8*	-1.7**	-1.8*
TRINITY_DN672_c0_g1	-1.7**	-1.9*	-0.7**	0.8
TRINITY_DN11995_c0_g1	-2.0**	0.2	-1.3**	0.7
