# Supplementary figures and images for: Novel Candidate Genes Differentially Expressed in Glyphosate-Treated Horseweed (Conyza canadensis)
Source: Genes (Basel). 2021 Oct 14;12(10):1616. doi: 10.3390/genes12101616 (PMC8535903; doi:10.3390/genes12101616)

0.84kg acid equivalent ha<sup>-1</sup>  
Round-up WeatherMax,  
24 h after spray

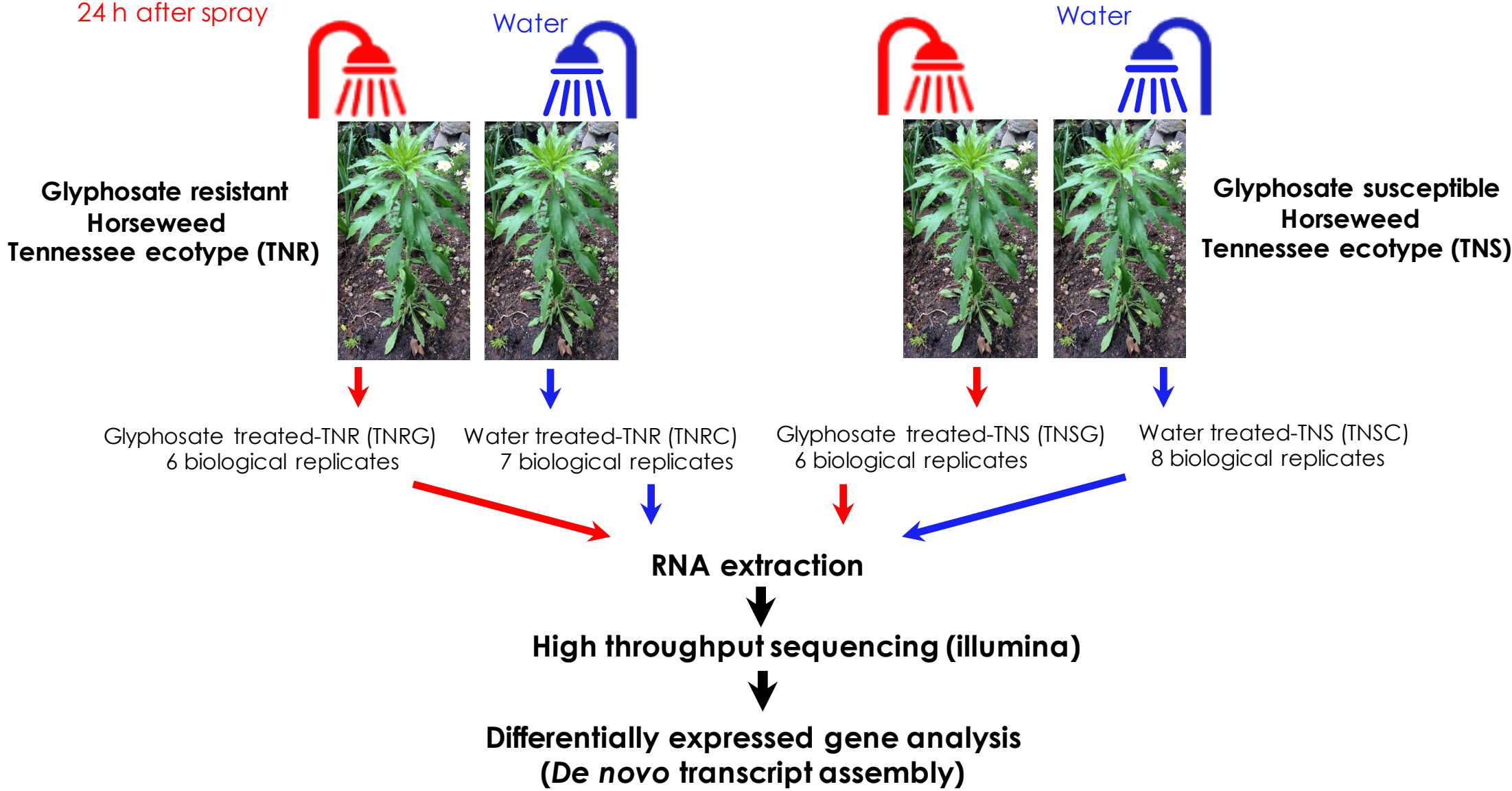

Supplement: Supplementary file 1 [file genes-12-01616-s001.zip › genes-1360991-supplementary/Figure S1.pdf]
